# Acoustofluidic Plasmapheresis System Designed for Ultralow Blood Volume Applications

**DOI:** 10.1021/acs.analchem.5c04042

**Published:** 2026-01-06

**Authors:** Amal Nath, Sara Marie Larsson, Andreas Lenshof, Wei Qiu, Thierry Baasch, Linda Nilsson, Thomas Thymann, Stanislava Pankratova, Magnus Gram, David Ley, Thomas Laurell

**Affiliations:** 1 Department of Biomedical Engineering, 5193 Lund University, Lund SE-223 63, Sweden; 2 Clinical Chemistry, Hospitals of Halland, Varberg SE-432 37, Sweden; 3 Department of Clinical Sciences Lund, Pediatrics, Lund University, Lund SE-221 84, Sweden; 4 Comparative Pediatrics, Section for Biomedicine, Department of Veterinary and Animal Sciences, University of Copenhagen, Frederiksberg DK-1870, Denmark; 5 Department of Neonatology, Skåne University Hospital, Lund SE-222 42, Sweden; 6 Department of Biomedical Science, Faculty of Health and Society, Biofilms-Research Center for Biointerfaces, Malmö University, Malmö SE-205 06, Sweden

## Abstract

We present an integrated acoustofluidic plasmapheresis system designed for ultralow blood volume applications, such as neonatal care, enabling in-line sampling and plasma separation from whole blood. The system combines a two-stage acoustophoresis chip with microperistaltic pumps and PDMS-based flow pulsation dampeners to ensure continuous, stable operation. The input whole blood is acoustically separated into a cell-free plasma fraction and a returnable cell fraction, enabling closed-loop operation for neonates who have a circulating blood volume as low as 50 mL. The system achieves a plasma generation rate of 27.5 μL/min with ∼100% cell removal, outperforming previous microfluidic plasma separation approaches in terms of purity, throughput, and minimal sample volume. Plasma quality was validated by quantifying hemolysis and residual cellular content, while system robustness was demonstrated across hematocrit levels up to 50%, which is close to the average upper hematocrit value in neonates. Compared to previous microfluidic techniques, our system achieves the fastest generation of clinical quality undiluted plasma with the lowest required blood volume, making it highly suitable for point-of-care integration in neonatal intensive care units.

## Graphical abstract

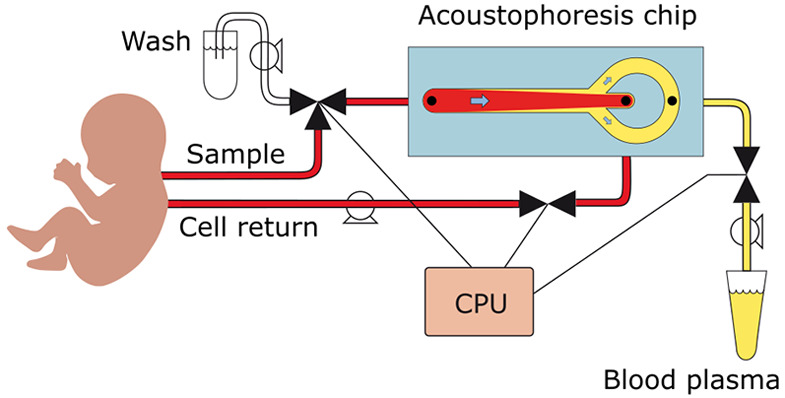

## Introduction

Neonatal intensive care is often required immediately after birth for many infants due to complications such as prematurity, congenital malformations, infections, or other serious health conditions. Continuous monitoring of the physiological status of the neonate is critical in early stages of life, and this is frequently achieved through routine blood testing. These diagnostic tests rely on the collection of blood samples at regular intervals, followed by plasma separation using centrifugation and subsequent clinical chemistry analysis. The current standard blood sampling procedures are however not well-suited to the unique physiology of neonates. These methods are largely adapted from sampling routines developed for adults with mature cardiovascular systems, which do not account for the lower circulating blood volume. In particular, preterm infants can weigh as little as 0.5 kg and may have total circulating blood volumes as low as 50 mL.^1^ Pediatric blood collection tubes, commonly used in clinical settings, typically draw around 0.5 mL of blood per sample. This can amount to nearly 1% of the entire circulating blood volume of a preterm neonate - a significant loss, especially considering the need for frequent monitoring.

Despite advances in laboratory instrumentation that allow many diagnostic tests to be performed on microliter-scale plasma volumes, the current sampling routines often still involve overcollection, i.e. collecting significantly larger blood volumes than necessary.^2^ This practice not only leads to the unnecessary loss of endogenous blood but also increases the risk of several serious complications. For example, iatrogenic anemia is a common consequence of excessive blood sampling in neonates, which leads to increased blood transfusion needs.^3^ Furthermore, sampling-related blood loss has been associated with the development of chronic conditions such as bronchopulmonary dysplasia and retinopathy of prematurity.^4–6^ An additional consequence of the current sampling routines is the increased susceptibility to infections, as manual sampling increases the risk of introducing pathogens. Therefore, minimizing sampled blood volume and reducing manual intervention are essential in improving the safety of neonatal blood testing.

Microfluidics-based blood sampling and separation systems are well suited to low blood volume settings and involve low flow dead volumes, offer easy downstream integration to point of care analyzers, and can be integrated into existing connected catheters. Acoustofluidics has emerged as a particularly promising approach for gentle, label-free, noncontact manipulation of biological components within fluids.^7–10^ Previous studies have demonstrated its utility in the focusing, separation, and trapping of cells,^11–14^ bacteria,^15–17^ and extracellular vesicles.^18,19^

To address the challenge of blood loss during sampling in settings such as neonatal care units, a potential solution is to use an acoustofluidics-based in-line blood sampling system (Figure 1A). In the system proposed in this work, each sampling cycle operates as a closed-loop process: a small volume of blood is continuously drawn from the neonate into the device, where acoustic forces separate it into two fractions. The plasma fraction is collected for diagnostic tests, while the remaining cell fraction containing red blood cells, white blood cells, and platelets are simultaneously returned to the neonate, thereby minimizing overall blood loss. The system is designed to operate as a compact, automated platform located within the neonatal incubator, thereby reducing the need for manual handling and minimizing the risk of infection.

**Figure 1:**
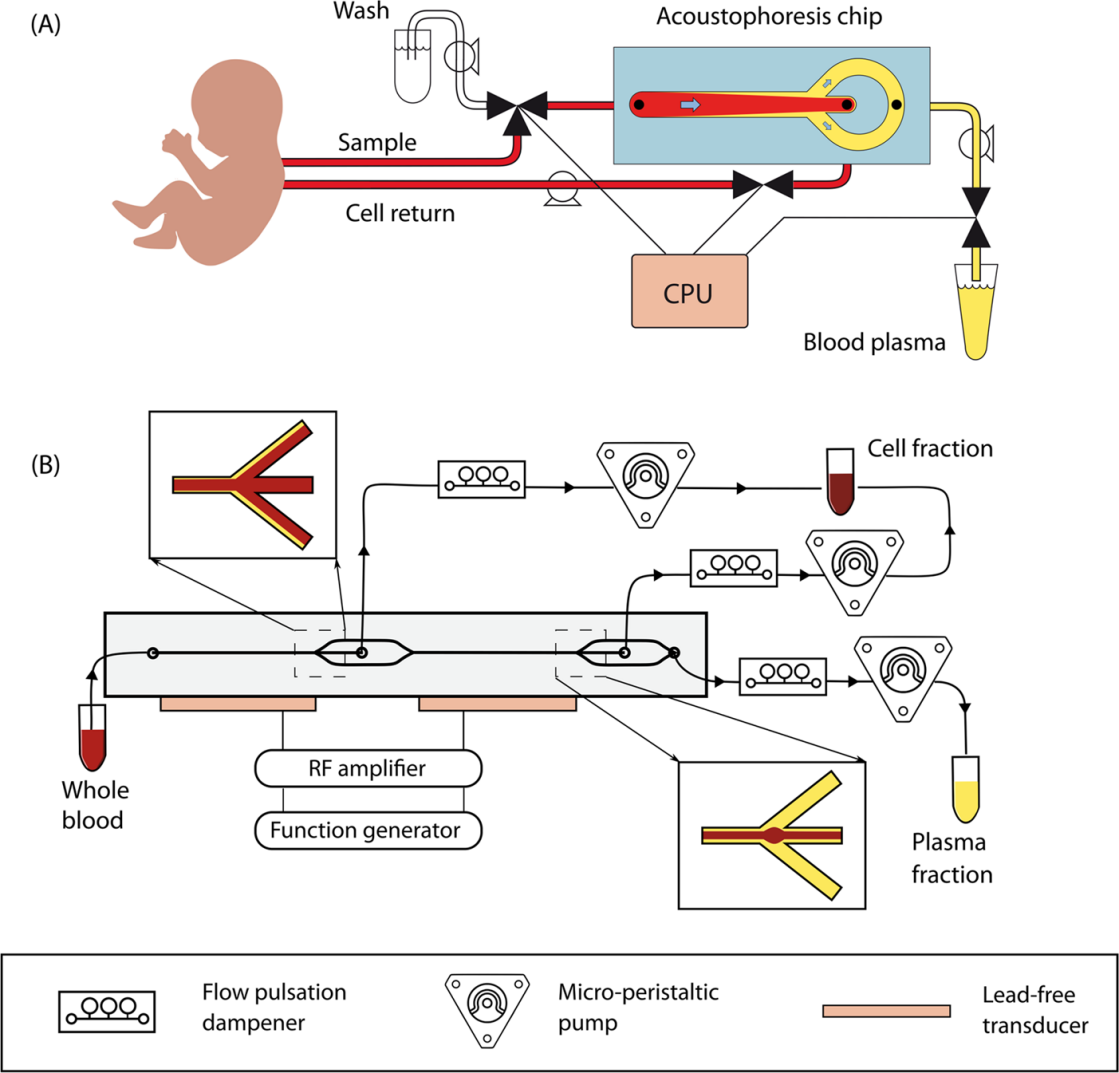
(A) Design of an integrated plasmapheresis system for neonatal care in which blood cells are separated and can be returned to the infant while plasma is collected for diagnostic tests. (B) Implementation of the design with adult donor blood obtained in blood collection tubes.

This prototype study presents the microfluidic design and performance evaluation of an acoustofluidics-enabled blood separation system. A key aspect of system integration is the selection of a suitable fluid pumping mechanism. In microfluidics, flow is commonly driven either by syringes or pressure systems. The use of syringe-based pumping poses challenges in effectively purging whole blood residues after each sampling cycle without extensive buffer washing. Pressure-driven systems, while continuous, raise safety concerns when used in online setups directly coupled to infants. These limitations motivated the use of peristaltic pumping, which is widely used in clinical online blood handling systems. Since acoustophoretic cell focusing is sensitive to flow rate, pulsation dampeners were integrated to manage the inherent oscillations of peristaltic flow, ensuring stable performance tailored to the fluid dynamics of the system.

Initial validation of the acoustofluidic blood separation system was conducted to assess plasma separation efficiency, using blood samples from healthy adult donors as a model system. The results demonstrate improved performance compared to existing microfluidic separation techniques, both in terms of the blood volume required to generate sufficient plasma for diagnostic testing, and also the time required for the separation process. The purity of the separated plasma was assessed by measuring residual cell counts. In addition, the integrity of the cell fraction to be returned to the neonate was evaluated based on clinical guidelines for hemolysis,^20,21^ ensuring that the procedure did not damage the erythrocytes during processing. Additionally, the separation performance was evaluated over a range of hematocrit (Hct) levels (40%–50%) to evaluate the robustness of the system. Finally, a preclinical evaluation using blood samples from preterm piglets born by elective cesarean section, was conducted, confirming the compatibility of the system for future in vivo animal testing.

## Methods

### Working Principle

The acoustofluidic plasmapheresis system enables blood sampling, plasma separation, and return of cellular components. It comprises an acoustophoresis-based separation chip, three microperistaltic pumps, and flow pulsation dampeners for stable flow control. Plasma separation is achieved via acoustophoresis, where particles experience an acoustic radiation force in a one-dimensional half-wavelength standing wave field, directing them toward pressure nodes. The acoustic radiation force acting on a spherical particle is expressed as^22^

1 $$F_{\mathrm{rad}}=4{\pi a}^{3}\phi \left(\mathrm{\kappa ̃},\mathrm{\rho ̃}\right){kE}_{\mathrm{ac}}\sin\left(2ky\right)e_{y}$$

where a is the particle radius, k is the wavenumber, E_ac_ is the acoustic energy density, y is the distance to the channel wall, and ϕ­(κ̃,ρ̃) is the acoustic contrast factor, dependent on compressibility (κ̃) and density (ρ̃) ratios between the particle and the medium. Large particles such as blood cells experience strong forces and are easily separated from plasma. Smaller particles like platelets display a lower acoustophoretic mobility, due to the volume dependence of the radiation force, and are thus more difficult to focus. A schematic of the experimental setup is shown in Figure 1B, with individual components described in the following sections.

### Separation Chip

The glass-based acoustofluidic separator consisted of two consecutive separation channels, each ending with trifurcations. The channels were isotropically etched and had cross sectional dimensions 440 μm × 150 μm and lengths 34.4 mm and 31.6 mm. Lead-free (Bi,Na)­TiO_3_-BaTiO_3_-(Bi,Na)­(Mn,Nb)­O_3_ (BNT-BT-BNMN) transducers (dimensions: 25 × 2 × 1.2 mm^3^) developed by Honda Electronics (HC-70BN, Honda Electronics, Toyohashi, Japan) were glued to the first and second channels at the sides. The selection of lead-free piezoelectric transducers was motivated by their superior performance compared to conventional lead zirconate titanate (PZT) transducers,^23^ as well as their high biocompatibility. The actuation of the transducers created an acoustic standing half-wave field inside the channels. The resulting acoustic radiation forces, directed toward the pressure node at the center, enabled complete removal of blood cells from whole blood over the two separation stages. In the first stage, the majority of blood cells from whole blood were focused and removed at the end of the trifurcation through the first central outlet. The remaining blood proceeded to the second stage with a much lower concentration where the rest of the cells were focused and removed through the second central outlet, providing clean plasma through the side outlet. A two-stage separation design was chosen because, in a single-stage configuration, the focused stream of blood cells occupies a large part of the channel width, owing to the large concentration of cells in the input blood. This limits the flow rate at which cell-free plasma can be extracted from the chip at the side outlet. Moreover, in a single-stage setup, minor fluctuations in flow rate can lead to cell spillover into the side outlet. By contrast, the two-stage configuration operates with a reduced cell concentration in the second stage, enabling focusing of blood cells into a narrower stream and improving the stability and purity of plasma extraction.

### Acoustic Actuation

The transducers were driven by a dual-channel function generator (AFG 3022B, Tektronix, Inc., Beaverton, Oregon) and an in-house designed dual-channel amplifier. The two transducers were excited by sinusoidal signals with a linear frequency sweep (sweep duration: 1 ms) between 1.940 MHz – 1.946 MHz and 1.934 MHz – 1.940 MHz, with constant peak-to-peak voltages of 22.2 V and 19.8 V respectively. The use of frequency sweep instead of a single frequency served two purposes. First, it enabled the focusing of cells across blood samples from donors with varying Hct values and compositions. Second, frequency sweep additionally ensured that irregularities in acoustic focusing across the length of the channel were smoothened out.^24^ The difference in optimal operating frequencies between the two transducers primarily due to variations in the glue layer thickness caused by manual bonding of the transducers, as well as slight differences in the channel media, since the blood in the second channel had a lower cell concentration compared to the first channel. The voltage applied to the transducer was constrained by the maximum allowable heating of the chip. The voltage was thus set to maintain the temperature of the chip below 34 °C, a few degrees lower than the physiological temperature of 37 °C, to ensure that the quality of the cells or the generated blood plasma did not degrade due to heating.

### Pumps and Flow Settings

DC motor-driven microperistaltic pumps (Takasago Fluidic Systems, Nagoya, Japan) were connected at the three outlets operating in suction mode to draw the blood into the plasmapheresis chip. Peristaltic pumps were chosen because of their safe and reliable operation in existing medical applications, including apheresis and dialysis instrumentation. The voltages applied to the DC motors were to tuned to set the flow rates at the first and second central cell fraction outlets to 55 μL/min each and the side plasma fraction outlet to Q_*p*_ = 27.5 μL/min, which resulted in a system inlet flow rate of Q_*i*_ = 137.5 μL/min. This corresponded to a plasma flow ratio of Q* = 100 × Q_*p*_/Q_i_ = 20%. Between two successive experimental runs, the flow path was cleaned with a cleaning procedure that introduced BD FACS Clean (10% bleach), BD FACS Rinse solutions (BD Biosciences, San Jose, CA) and Milli-Q water into the system sequentially. The system was then primed with normal saline (0.9% NaCl) before operation. This cleaning procedure was adopted as the donor blood samples were obtained at intervals of several days and the experiments were conducted under nonsterile conditions. In a clinical setting, the system as well as the cell return loop can be purged into a waste with normal saline alone.

### Flow Pulsation Dampeners

Periodic fluctuations arising due to the pulsatile nature of microperistaltic pumps necessitated the use of flow pulsation dampeners to stabilize the flow. Here, we used a passive flow stabilization technique which made use of air compliance chambers.^25,26^ The flow dampener, fabricated using soft lithography in polydimethylsiloxane (PDMS), consisted of three cylindrical air chambers transverse to a 400 μm wide and 300 μm deep main channel distributed along a length of 30 mm. The pressure pulses in the flow were dampened by the compression and relaxation of air in these chambers, which resulted in a smoother fluid flow. Different air chamber volumes were evaluated to achieve desired reduction in fluctuation amplitudes at the lowest fluctuation frequency under operation of the microperistaltic pumps. The dimensions of the dampeners are provided in Table S1 in Supporting Information.

### Blood Samples

Blood samples from healthy adult donors were drawn by phlebotomists and collected in blood collection tubes (BD Vacutainer, Plymouth, UK) coated with lithium heparin (Li-Hep) anticoagulant. Blood was drawn into the system directly from the collection tube. Flow cytometry analysis of the collected blood plasma along with the input whole blood was performed using a BD FACS Canto II (BD Biosciences, San Jose, CA) cytometer. Prior to flow cytometry analysis, the blood and plasma samples were diluted 10000× and 100× respectively, in phosphate-buffered saline. For measuring platelet counts, fluorochrome-labeled monoclonal anti-CD61 antibodies (BD Biosciences, San Jose, CA) were added and incubated for 20 min to bind to the platelets. Flow cytometry events were recorded at medium flow rate (60 μL/min) for 1 min. Hemoglobin concentration in the samples was measured using a HemoCue Plasma/Low Hb System (HemoCue AB, Ängelholm, Sweden). Hematocrit in the samples were measured using a hematocrit centrifuge (Hematokrit 210, Hettich GmbH, Germany).

For experiments with animal blood samples, porcine blood was collected from three different sources: adult sows (Danish Landrace × Yorkshire), umbilical cord blood at the time of delivery, and preterm piglets (Danish Landrace × Yorkshire × Duroc) at postnatal day 1. The preterm piglets were delivered via cesarean section at a gestational age of 106 days, corresponding to approximately 90% of full term gestation. Blood collection was carried out under an approved ethical protocol (license no.: 2020-15-0201-00520) and performed by trained veterinary personnel. For adult sows and preterm piglets, blood was drawn using sterile syringes by venipuncture. In the case of umbilical cord blood, samples were collected by gently milking the cord to extract residual blood, ensuring minimal coagulation and hemolysis. All collected blood samples were immediately transferred into lithium heparin (Li-Hep) anticoagulated blood collection tubes.

## Results

### Performance of the Flow Pulsation Dampener

The oscillations under operation of the pumps were observed to be in the frequency range f = 1–3 Hz. Therefore, the pulsation dampener needed to be designed to effectively damp out oscillations at the lowest frequency, f = 1 Hz. The performance of the pulsation dampener was evaluated for a total of eight different dampener dimensions (Table S1) using the experimental setup shown in Figure 2A, by measuring the decrease in fluctuation amplitude across the dampener. Milli-Q water was supplied to the inlet of the dampener using a syringe pump (neMESYS, Cetoni GmbH, Germany) at a sinusoidal volumetric flow rate defined as Q = 70 + 30 sin­(2πt) μL/min where Q is the instantaneous flow rate and t is time in seconds. Q was monitored at the inlet and outlet of the pulsation dampener with flow sensors (Flow unit, Fluigent, France). Figure 2B shows the deformation of the liquid–air interface due to compression and relaxation of air in the chamber. During flow transients in the main channel, an increase in flow rate (upsurge) drives the liquid into the side channel, resulting in compression of air within the chamber. Conversely, during a decrease in flow rate (downsurge), the compressed air expands, displacing the liquid back into the main channel and thereby modulating the pressure and flow dynamics. The effect of chamber volume on the dampening of flow is shown in Figure 2C for three different chamber volumes. Larger air chambers provide more compliance to the system, and the air can undergo greater compression and relaxation, thereby resulting in a more uniform flow at the outlet. The pulsation dampener was also modeled using hydrodynamic circuit analysis as shown in Figure 2D. The channels were modeled as linear resistive elements, while the air chamber was represented by a capacitive element, forming an RC low-pass filter analog in the hydraulic domain. The frequency-domain transfer function describing the ratio of output to input flow pulsation amplitudes was given by

2 $$\mathrm{TF}=\frac{1+R_{M}C_{h}i\omega }{1+\left(R_{M}+R_{S}\right)C_{h}i\omega }$$

where R_M_ and R_S_ denote the hydraulic resistances in the main and side channels respectively, C_h_ is the hydraulic compliance of the air chamber, and ω = 2πf is the angular frequency. The frequency f = 1 Hz, corresponds to the lowest frequency of pulsations during operation. The hydraulic compliance of the air chamber was computed using the isothermal compressibility assumption C_h_ = V_ch_/P_0_, where V_ch_ is the air chamber volume and *P*_0_ = 10^5^ Pa is set to ambient pressure.^25^ The magnitude of the transfer function |TF| was evaluated at eight different air chamber volumes. The transfer function magnitude from the model was compared with the experimental results. Since the pulsation dampener comprised an array of three air chambers, the total attenuation of the pulsation amplitude was the cube of the single-chamber transfer function magnitude, i.e., |TF|^3^. The attenuation obtained from the model was compared to the experimentally observed attenuation, A_o_/A_i_,where A_i_ and A_o_ denote the input and output flow pulsation amplitudes, respectively. From the flow sensor measurements, A_o_/A_i_ was computed by performing a Fast Fourier Transform of flow rate vs time (Q vs t) signals at the inlet and the outlet of the dampener. The comparison between the model predictions and experimentally obtained values, shown in Figure 2E, indicates a good agreement. The use of larger air chambers led to higher pulse attenuation in flow. The required air chamber volume can be determined considering the size of the dampener and the desired level of damping. Here, pulsation dampeners with air chambers with V_ch_ = 97 μL were used. Figure 2F presents the performance of the dampener when used with a microperistaltic pump. The pump was operated at 47 μL/min, corresponding to flow oscillations of f ≈ 3 Hz. As expected, higher-frequency flow oscillations are damped more effectively by the dampener, resulting in a better stabilized flow. The flow pulsation amplitude reduced from 19.9 μL/min to 0.7 μL/min, a 96.5% reduction.

**Figure 2:**
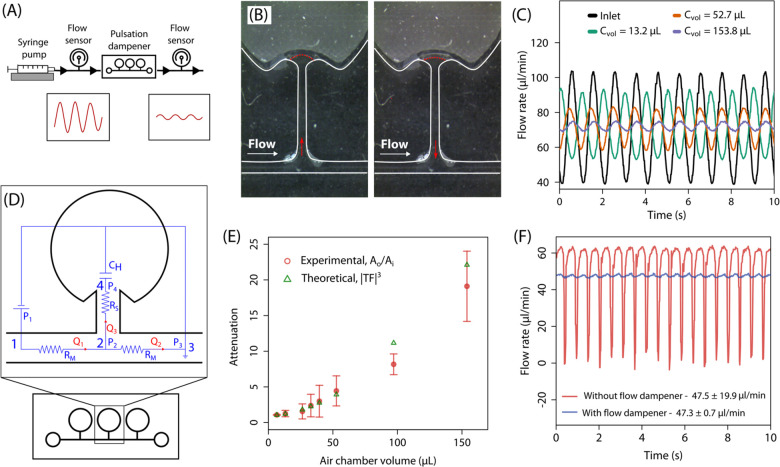
(A) Experimental setup used to quantify the performance of the pulsation dampener. Inset shows instantaneous flow rate vs time measured by the flow sensor. (B) Experimental images capturing the dynamic evolution of the liquid−air interface as the liquid enters the side channel during flow rate upsurge and exits during downsurge (see Supplementary Video SV1). Red dotted lines depict the liquid−air interface, and red arrows indicate the direction of flow into or away from the side channel during upsurge and downsurge. (C) Comparison of flow rate variations over time at the dampener inlet and outlet for three different chamber volumes. (D) Hydrodynamic circuit representation of the pulsation dampener, where channel segments are modeled as resistances R_M_ and R_S_ and the air chamber by compliance C_H_. (E) Experimentally measured attenuation compared to the theoretical values for different chamber volumes. Mean ± SD is reported. *n* = 3. (F) Flow rate measured over time from a microperistaltic pump with and without a pulsation dampener.

### Focusing of Blood Cells

The high concentration of cells in whole blood required a two-stage process for effective removal of blood cells. The use of pulsation dampeners was critical in achieving efficient cell separation to generate diagnostic plasma. As shown in Figure 3A, in the absence of a pulsation dampener to stabilize the flow, periodic fluctuations caused focused cells to spill into the stage II side outlet. This disrupted the separation process and lowered the purity of the plasma fraction. In contrast, Figure 3B illustrates how the dampener stabilized the flow, ensuring controlled cell removal at the end of both separation stages. At the end of the first stage, most cells were removed through the central outlet, while the remaining sample with a lower cell concentration flowed into the second stage. The reduced concentration allowed the cells to focus into a narrower stream, which enabled complete removal. In the second stage, flow rates were set so that the focused stream of cells did not entirely cover the cell outlet. Instead, a thin layer of plasma separated the cells from the walls of the central outlet. This flow setting improved system robustness, preventing small flow fluctuations from causing cell spillover into the plasma outlet. To assess the degree of focusing in the two separation stages, Hct was measured in both the input whole blood and in the cell fractions collected separately at the first and second cell outlets (Figure 3C). For input whole blood of Hct – 41.7 ± 1.6, the cell fraction collected at the first outlet had Hct – 77.0 ± 4.1, corresponding to nearly a 2-fold concentration, while at the second outlet the measured Hct was similar to that of input blood – 42.0 ± 6.6.

**Figure 3:**
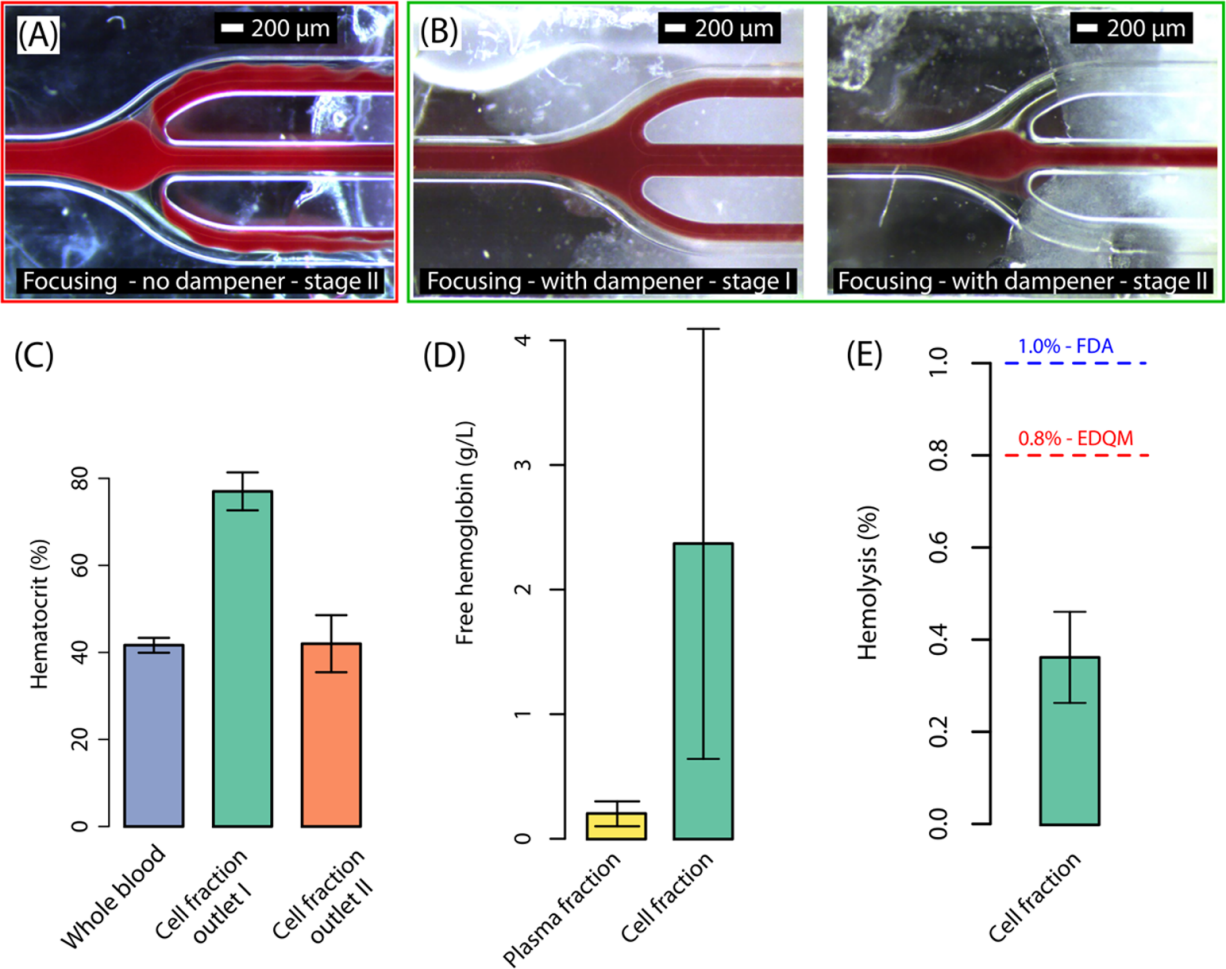
(A) Focusing of blood cells without the dampener leads to spillover of cells into the stage II plasma outlet due to pulsations in flow (see Supplementary Video SV2). (B) Flow dampener ensures stable flow leading to efficient cell removal (see Supplementary Videos SV3 and SV4). Scale bar: 200 μm. (C) The hematocrit of the input whole blood and the collected cell fraction at the outlets. (D) The concentration of free hemoglobin in the plasma fraction and cell fractions collected at the first and second central outlets. (E) Hemolysis in the cell fractions. Mean ± SD is reported. *n* = 3.

### Free Hemoglobin and Hemolysis

To assess plasma quality and the gentleness of separation, the degree of cell lysis was quantified by the measuring the free hemoglobin (fHb) concentration in the separated cell and plasma fractions respectively (Figure 3D). fHb concentration in the separated plasma fraction was found to be 0.2 ± 0.1 g/L. For diagnostic measurements in plasma, fHb concentrations below 0.27 g/L have been previously shown not to cause any significant interference with a majority of commonly ordered biochemical parameters.^27^ To assess the potential for returning intact cells to the infant, the fHb concentration in the cell fraction was also evaluated. Compared to the plasma fraction, the measured fHb concentration in the cell fraction was seen to be higher: 2.4 ± 1.7 g/L. Hemolysis (H) in the cell fraction was computed by comparing fHb concentration to the total hemoglobin (tHb) concentration, corrected for hematocrit, using the following equation^28^

3 $$H\left(\%\right)=\left(100-\mathrm{Hct}\right)\times \left(\frac{\mathrm{fHb}}{\mathrm{tHb}}\right)$$

The European Directorate for the Quality of Medicines and HealthCare (EDQM) sets an upper limit of H = 0.8% in red blood cell (RBC) products intended for transfusion.^20^ While the U.S. Food and Drug Administration (FDA) has not defined a formal limit for standard RBC products, it recommends a maximum of H = 1.0% hemolysis for deglycerolized RBCs.^21^ The hemolysis level in the cell fraction was measured to be 0.37 ± 0.10%, well within both reference thresholds (Figure 3E).

### Cell and Platelet Separation Efficiency

In neonates, Hct varies with both gestational age and postnatal age. In particular, preterm neonates exhibit a wide range of Hct values over the first 28 days of life, with average Hct (95% intervals) between 33% (24%–45%) and 49% (37%–61%).^29^ Therefore, it is important to evaluate system performance with input blood samples spanning a broader Hct range than in healthy adult blood samples. To investigate the influence of Hct on separation performance, the efficiency of the system in removing cells and platelets was assessed at three different Hct levels. The hematocrit of healthy adult blood was adjusted postcentrifugation to 40%, 45%, and 50% by removing plasma and resuspending the cells to achieve uniform cell concentration at each target Hct level. Hct levels below 40% were not assessed as cell and platelet removal is less challenging at lower concentrations. The separation efficiency was defined as

4 $$E=1-\frac{N_{p}}{N_{i}}$$

where N_p_ and N_i_ were the number of cells (or platelets) per μL in the collected plasma and input whole blood samples, respectively. For each target level, plasma flow ratio was varied to evaluate separation performance over a range of Q*. This was achieved by maintaining a constant flow rate for the cell fraction while increasing the plasma flow rate, thereby varying Q* from 15% to 33%. Figure 4A shows the separation efficiency of cells (E_c_ and platelets (E_p_ across different Hct levels and plasma flow ratios (Q*. In general, both E_c_ and E_p_ decreased with increasing Hct, and the difference was more pronounced at higher Q*. This trend can be attributed to the increased red blood cell concentration at higher Hct, which enhances the possibility of cells spilling into the plasma outlet (Figure 4B). At Hct levels of 40% and 45%, nearly 100% cell separation efficiency (E_c_ ≈100%) was maintained at Q* ≤ 25%, while platelet separation efficiency (E_p_) remained around 90%. However, at Hct = 50%, cell-free plasma (E_c_ ≈ 100%) was achieved only up to Q* = 20%, with separation performance dropping off rapidly as Q* increased. The decrease in E_p_ was even more pronounced at elevated Hct, particularly beyond Q* = 25% For instance, at 45% Hct and Q* = 33%, E_p_ was only 67.2 ± 15.5%, indicating that more than 30% of the platelets from the input sample were collected in the plasma outlet. Therefore, to ensure minimal contamination from residual cells and platelets in plasma at higher input blood Hct levels, the system needs to be operated at lower Q_*p*_.

**Figure 4:**
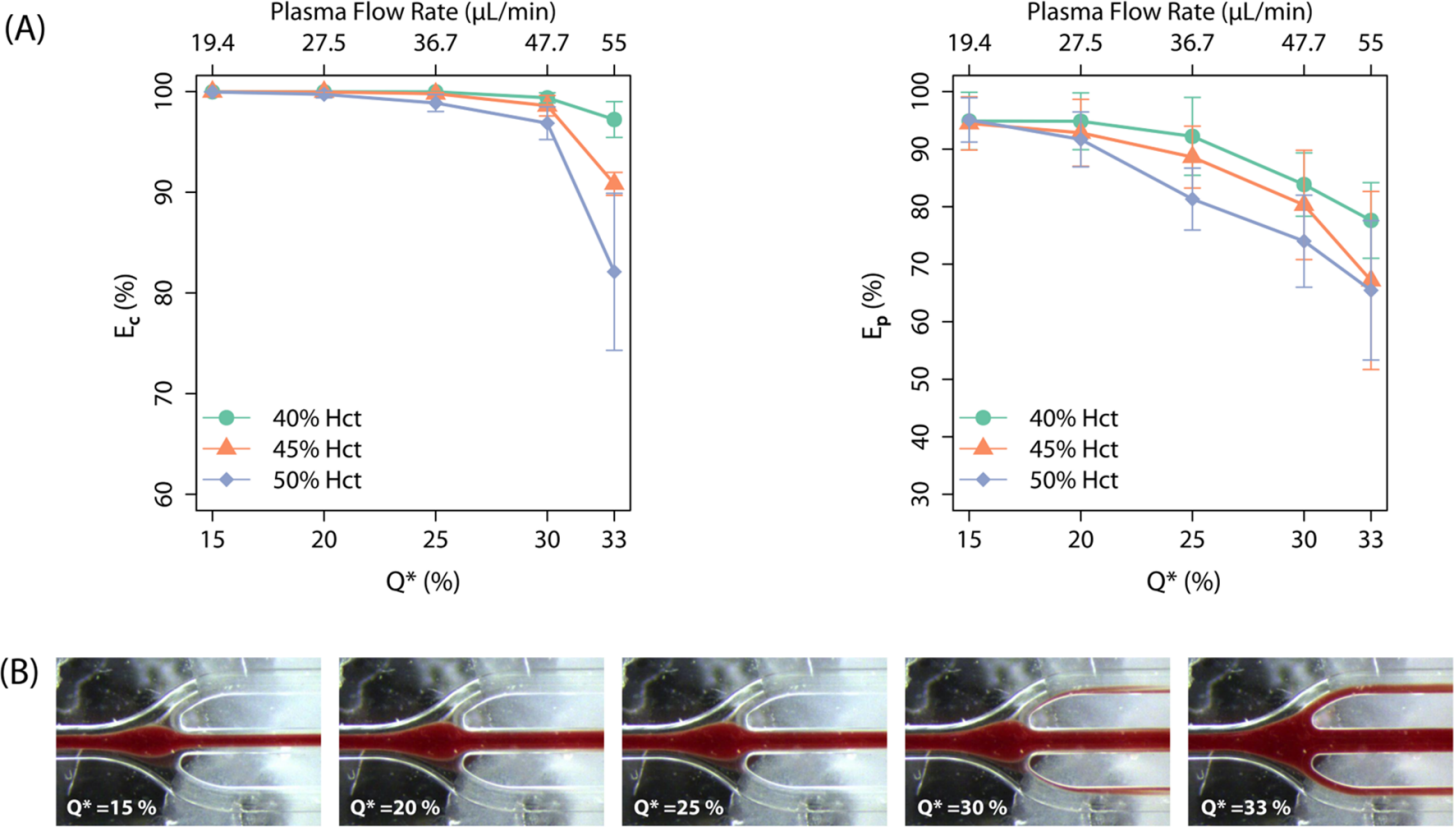
(A) Variation in cell separation efficiency (E_c_) and platelet separation efficiency (E_p_ with plasma flow ratios (Q*) at different hematocrit (Hct) (Mean ± SD, *n* = 3). The corresponding plasma flow rates are included at the top of the plots. (B) Experimental images at the end of second stage, showing the spillover of cells into the plasma outlet at high Q*.

### Comparison to Previous On-Chip Separation Techniques

Rapid generation of clean cell-free blood plasma is important for continuous diagnostic monitoring. In Table 1, the results from the present study are compared to previous passive and active techniques for microfluidic blood plasma separation from whole blood. The cell separation efficiency of ∼100% achieved in this study is comparable to the highest reported values. A comparison with other techniques is also made for quality requirements proposed by the EDQM for fresh frozen plasma (<6000 RBCs per μL). The plasma generation parameters were compared with respect to generation of 200 μL of plasma, which is the commonly required volume to perform a set of routinely ordered clinical chemistry tests on a modern analyzer platform. The present study achieved the fastest time to collect 200 μL of undiluted plasma (6.4 min) with the lowest whole blood volume required (800 μL), in comparison to previous studies which show separation efficiencies of ∼100%.

**Table 1:** Comparison with Other Microfluidic Techniques for Separation of Blood Plasma from Undiluted Whole Blood

								Generation of 200 μL undiluted plasma	
Ref.	Method	Type of pumps	Hct (%)	Plasma flow ratio Q* (%)	Whole blood throughput (μL/min)	Cell separation efficiency (%)	Within EDQM requirements (<6000 RBCs per μL)?	Time (minutes)	Blood volume (mL)
^30^	Hydrodynamic	Syringe pump	45	n/a	500	98.5	No	n/a	n/a
^31^	Hydrodynamic	n/a	45	12	33.3	97	No	50.1	1.7
 ^32^	Acoustofluidic	Microperistaltic pump	45	n/a	20	85–95	No	n/a	n/a
^33^	Acoustofluidic	Syringe pump	45	12.5	80	∼100	Yes	20	1.6
 ^34^	Acoustofluidic	Syringe pump	45	20	50	∼100	Yes	20	1
 ^35^	Hydrodynamic	Syringe pump	40–50	9	50	99.96	Yes	45	2.2
 ^36^	Hydrodynamic	Syringe pump	45	5	33.3	99	No	120	4
 ^37^	Hydrodynamic	Syringe pump	45	10	250	64	No	8	2
Present study	Acoustofluidic	Microperistaltic pump	45	25	125	∼100	Yes	6.4	0.8

### Transition to in Vivo Animal Testing

As a preliminary step toward in vivo validation, the separation performance of the acoustofluidic plasmapheresis system was assessed using freshly collected porcine blood samples. The preterm piglet model (Figure 5A) was selected due to its well-established relevance to preterm human infants, given their similar size, developmental physiology, and hematological parameters.^38^ Two independent samples from each of the three sources (adult sows, umbilical cord at the time of delivery, preterm piglets) were assessed. The plasma separation protocol and quality assessment tests were the same as those performed for human blood samples. As shown in Figure 5B, the hematocrit in the sow blood and umbilical cord blood samples were similar with Hct <30% for all samples. In contrast, the piglet blood samples exhibited more variability with Hct = 25% and 38%, likely indicative of differences arising from gestational period and individual physiology. Notably, Hct values were lower than that for adult human samples, and therefore the system could be operated at higher Q* without spillover of cells into the plasma outlet (i.e., cell separation efficiency E_c_ ≈ 100%). For sow blood and cord blood samples, the system generated cell-free plasma at a whole blood flow rate of 156 μL/min with a corresponding plasma flow rate of 46 μL/min (Q* = 29.5%). Cell-free hemoglobin (fHb) concentration in the separated plasma fraction (Figure 5C) was found to be comparable to fHb concentration in plasma obtained by traditional centrifugation at 2000g × 10 min. The fHb levels were also similar to those measured in plasma fraction separated from human blood samples. The degree of hemolysis in the collected cell fraction (Figure 5D) was found to be lower than the regulatory threshold (H < 0.8%) specified for human RBC products. This indicates that the system maintains cell integrity during processing, similar to performance using human blood samples. These initial experiments validated the performance of the system with animal blood samples and indicated its readiness for in vivo studies.

**Figure 5:**
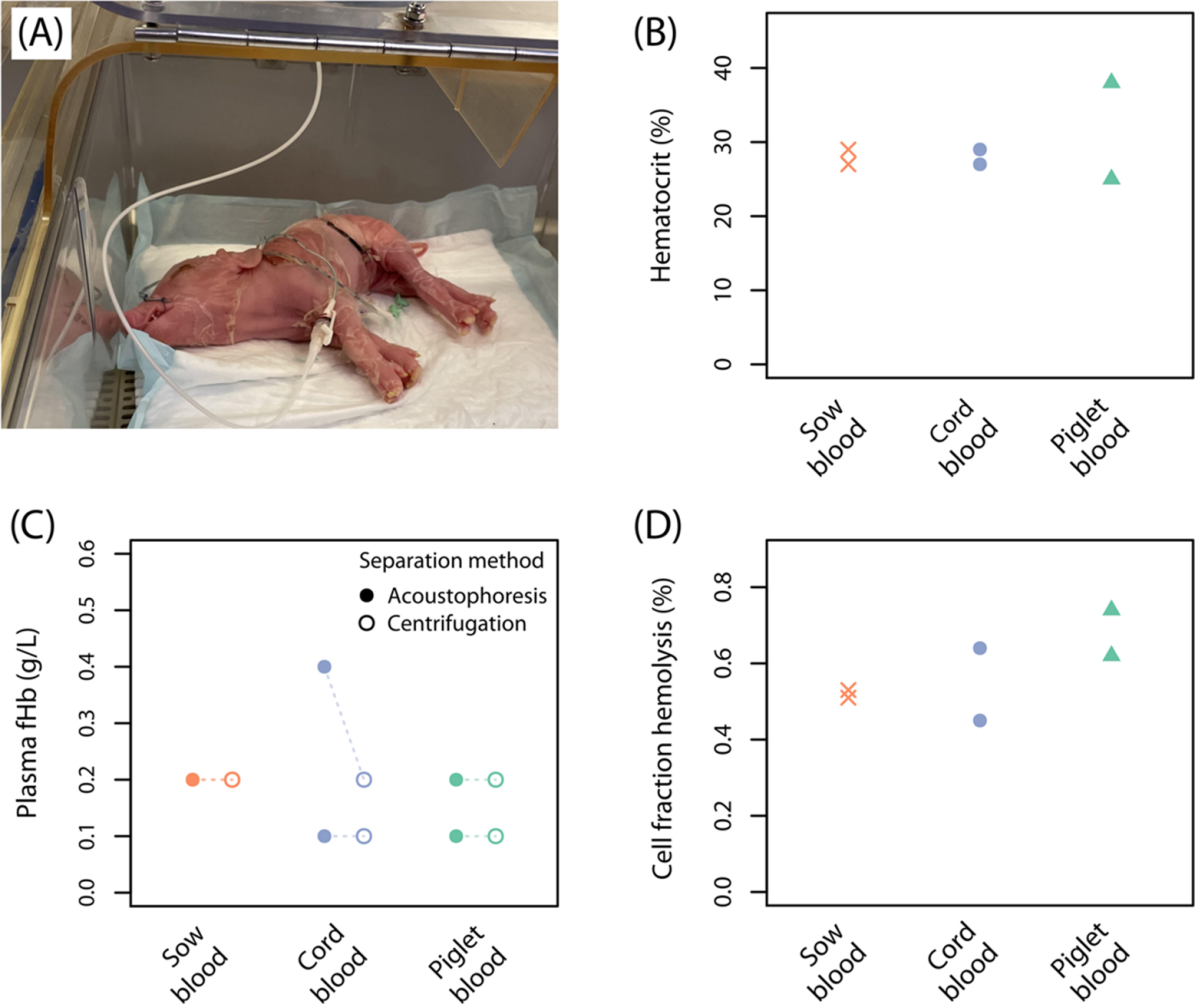
(A) Preterm piglets serve as excellent models for preterm human infants due to comparable body size and organ development. (B) Hematocrit values measured in the whole blood samples collected from sows, umbilical cord at the time of delivery, and preterm piglets. (C) Cell-free hemoglobin (fHb) concentrations in the separated plasma fraction evaluated against fHb concentrations in plasma obtained from traditional centrifugation (2000*g* × 10 min). fHb concentrations in both sow blood samples were measured to be same. Faint dotted lines connect paired samples for comparison. (D) Hemolysis in the collected cell fraction was <0.8% for all samples indicating minimal cell damage during processing. Sample size: *n* = 2 for all groups.

## Discussion

The present study demonstrates a significant improvement in microfluidic blood plasma generation from undiluted whole blood compared to previous studies. The system differs from previous demonstrations of microsystem-based plasmapheresis in some key aspects. First, the system employs microperistaltic pumps, which are critical for continuous and safe returning of blood cells to the infant. In contrast, most prior studies that have demonstrated microfluidic blood plasma separation have relied on the use of syringe pumps, which operate in a stop-start manner and therefore cannot maintain continuous flow. Second, the herein presented system generates undiluted plasma with no significant hemolysis and close to 100% (>99.99%) cell separation efficiency, i.e. plasma is nearly cell-free. It has to be noted that separation efficiencies as high as 99% reported in previous studies still leaves approximately ∼10^7^ cells/mL in plasma when starting from ∼10^9^ cells/mL, highlighting the superior performance of the present system. High plasma purity is critical for diverse diagnostic tests, including coagulation assays such as PT (Prothrombin Time), aPTT (Activated Partial Thromboplastin Time), fibrinogen, and D-dimer. The presence of impurities, notably hemolysis, significantly compromises the sensitivity of these tests by causing optical or biological interference, in addition to their inherent sensitivity to dilution.^39^ Generation of undiluted plasma is also crucial for accurate measurement of proteins, enzymes, and hormones especially when present in low concentrations. Additionally, online dilution of blood is also not feasible as the cell fraction is to be returned to the neonate without risking circulatory volume overload. Third, the system demonstrates good separation performance at superior plasma flow ratios and consequently higher sampling flow rates and plasma generation rates than previous designs. In comparison to previous acoustofluidics-based systems that reported ∼100% cell separation efficiency,^33,34^ the performance enhancement in the present system primarily arises from two key design differences: (a) transducer placement and actuation approach, and (b) transducer material. In the current design, a side-actuation configuration is employed, wherein the transducer is attached to the chip’s sidewall to excite a standing-wave field across the channel width.^40^ This approach has proven significantly more efficient than the conventional bottom-actuation method, as it enables stronger acoustic fields with lower input power. Furthermore, the use of lead-free piezoelectric transducers (BNT-BT-BNMN) further improves device efficiency when combined with the side-actuation configuration, as reported by Qiu.^23^ Additionally, previous systems relied on longer microchannels and more complex syringe pump-based fluidics, which are unsuitable for continuous medical sampling. In contrast, the present system employs peristaltic pumps, providing a stable, compact, and reliable flow control method more ideal for clinical settings. Collectively, these advancements make the system potentially suitable for clinical applications such as neonatal intensive care, where frequent blood sampling is required.

## Supplementary Material











## Notes

Overview photo of the chip and the microperistaltic pumps (Figure S1), schematic showing the design of the pulsation dampener (Figure S2), and tabulated details on the dimensions of the pulsation dampeners (Table S1) (PDF)
Dynamic change in the liquid-air interface inside the flow pulsation dampener - Video SV1 (MP4)
Spillover of blood cells into the plasma outlet due to pulsations in the flow - Video SV2 (MP4)
Stable focusing of blood cells enabled by flow pulsation dampeners - Video SV3 (MP4)
Stable focusing of blood cells enabled by flow pulsation dampeners - Video SV4 (MP4)
