# Supplementary material for: Acoustofluidic Plasmapheresis System Designed for Ultralow Blood Volume Applications
Source: Anal Chem. 2026 Jan 6;98(2):1252–61. doi: 10.1021/acs.analchem.5c04042 (PMC12824985; doi:10.1021/acs.analchem.5c04042)
Supplement: Supplementary file 1 [file ac5c04042_si_001.pdf]

SUPPORTING INFORMATION

Acoustofluidic Plasmapheresis System Designed for Ultra-Low Blood Volume Applications

Amal Nath[1], Sara Marie Larsson[2,3], Andreas Lenshof[1], Wei Qiu[1], Thierry Baasch[1], Linda Nilsson[3], Thomas Thymann[4], Stanislava Pankratova[4], Magnus Gram[3,5,6], David Ley[3], Thomas Laurell[1,*]

[1]Department of Biomedical Engineering, Lund University, Lund SE-223 63, Sweden

[2]Clinical Chemistry, Hospitals of Halland, Varberg SE-432 37, Sweden

[3]Department of Clinical Sciences Lund, Pediatrics, Lund University, Lund SE-221 84, Sweden

[4]Comparative Pediatrics, Section for Biomedicine, Department of Veterinary and Animal Sciences, University of Copenhagen, Frederiksberg DK-1870, Denmark

[5]Department of Neonatology, Skåne University Hospital, Lund SE-222 42, Sweden

[6]Department of Biomedical Science, Faculty of Health and Society, Biofilms-Research Center for Biointerfaces, Malmö University, Malmö SE-205 06, Sweden

*Corresponding author: Thomas Laurell, E-mail: thomas.laurell@bme.lth.se

Table of contents	Page
**S1. Overview photo of the acoustophoresis chip and micro-peristaltic pumps**	S2
**S2. Schematic of pulsation dampeners**	S3
**S3. Dimensions of pulsation dampeners**	S4

S1. Overview photo of the acoustophoresis chip and micro-peristaltic pumps

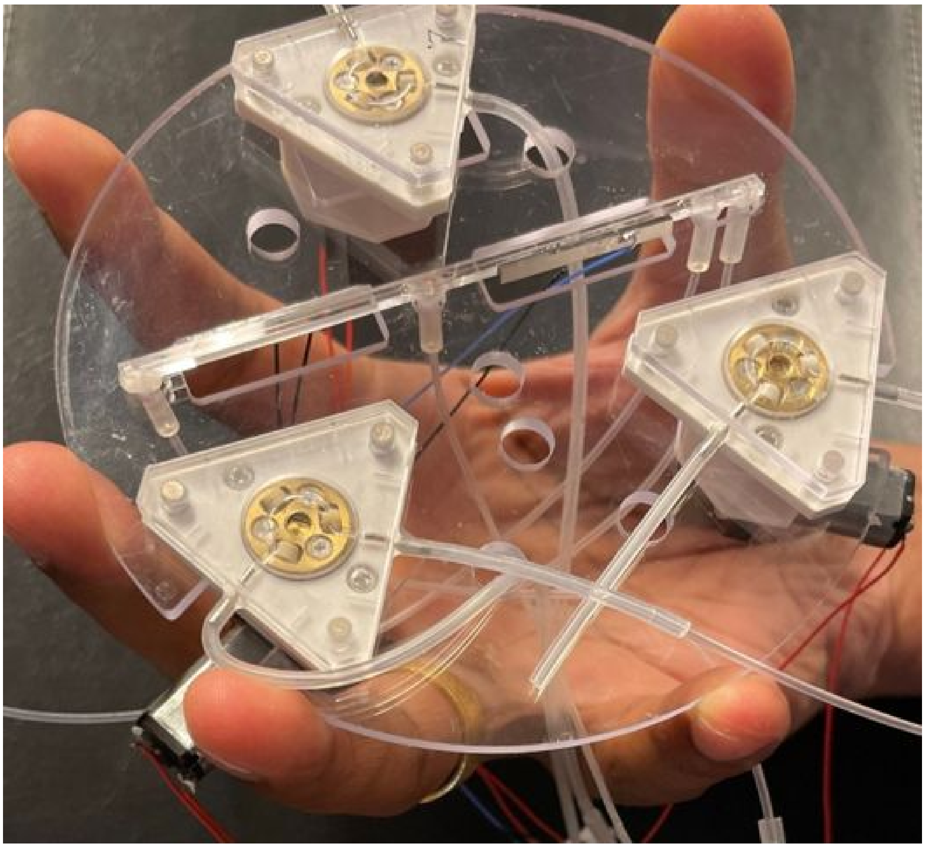

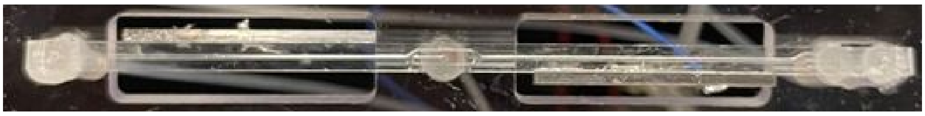

Fig. S1. The sampling and separation device comprises the acoustophoresis chip and three micro-peristaltic pumps. A zoomed-in image of the acoustophoresis chip is shown at the bottom.

S2. Schematic of pulsation dampeners

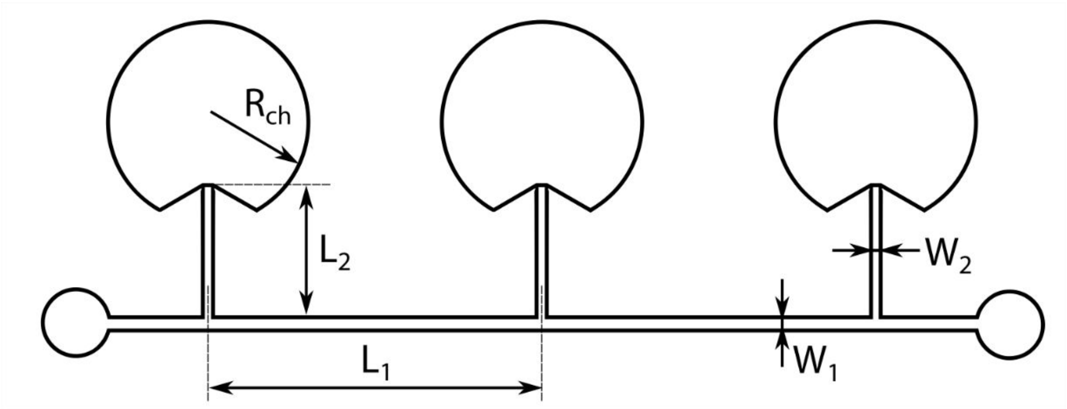

Fig. S2. Schematic with the design of the flow pulsation dampeners: Length between chambers along the main channel $L_{1,}$ Length of side channel L_2, Width of the main channel W_1, Width of the side channel W_2, Depth of channels H, Radius of air chamber R_{ch}, Depth of the air chamber H_{ch}, Volume of the air chamber $V_{ch.}$

S3. Dimensions of pulsation dampeners

Table S1: Dimensions of the dampeners used, with different air chamber volumes and channel lengths

	L_1	L_2	W_1	W_2	H	R_{ch}	H_{ch}	V_{ch}
D1	8 mm	4 mm	400 µm	300 µm	100 µm	3 mm	0.25 mm	6.6 µL
D2	8 mm	4 mm	400 µm	300 µm	100 µm	3 mm	0.50 mm	13.2 µL
D3	8 mm	4 mm	400 µm	300 µm	100 µm	3 mm	1.00 mm	26.4 µL
D4	8 mm	4 mm	400 µm	300 µm	100 µm	3 mm	1.25 mm	32.9 µL
D5	8 mm	4 mm	400 µm	300 µm	100 µm	3 mm	1.50 mm	39.5 µL
D6	8 mm	4 mm	400 µm	300 µm	100 µm	3 mm	2.00 mm	52.7 µL
D7	10 mm	4 mm	400 µm	300 µm	100 µm	4 mm	2.00 mm	97.1 µL
D8	12 mm	4 mm	400 µm	300 µm	100 µm	5 mm	2.00 mm	153.8 µL
